# Horse Behavior towards Familiar and Unfamiliar Humans: Implications for Equine-Assisted Services

**DOI:** 10.3390/ani11082369

**Published:** 2021-08-11

**Authors:** Lauren Brubaker, Katy Schroeder, Dawn Sherwood, Daniel Stroud, Monique A. R. Udell

**Affiliations:** 1 Animal and Rangeland Sciences, College of Agriculture, Oregon State University, Corvallis, OR 97333, USA; dawn.sherwood@oregonstate.edu (D.S.); monique.udell@oregonstate.edu (M.A.R.U.); 2 Animal and Food Sciences, College of Agricultural Sciences and Natural Resources, Texas Tech University, Lubbock, TX 79430, USA; Katy.Schroeder@ttu.edu; 3 Private Practice, Charleston, SC 29401, USA; strouddls@gmail.com

**Keywords:** equus, therapy horses, equine temperament, human–animal bond

## Abstract

**Simple Summary:**

Research on equine-assisted services (EAS) has traditionally focused on human benefits, while relatively little research has focused on the horse’s behavior and welfare. Therefore, this initial study aims to shed light on EAS horses’ behavior towards familiar and unfamiliar humans and how social behaviors might connect to EAS horse selection and retention. The results demonstrate that horses with no prior EAS experience show more interest in approaching familiar and unfamiliar people under certain test conditions compared to horses with more EAS experience. Interestingly, this social behavior did not appear to be linked to whether a horse was chosen for, or remained in, an EAS program. In addition, horse characteristics, such as startling at a new object, did not appear to influence selection and retention for EAS work. These findings indicate that EAS providers may have unique reasons for horse selection, and future research is needed to determine the specific characteristics of successful EAS horses.

**Abstract:**

While human benefits of animal-assisted therapy programs have been documented, relatively little research has been conducted on behavioral factors that predict a successful equine-assisted services (EAS) horse. This study compares the behavior of experienced and non-experienced EAS horses as well as horses selected for future EAS work in a series of sociability and temperament tests. No significant differences were found between experienced and non-experienced horses in the sociability measures or for most of the temperament tests; however, significant differences were found between groups in the brushing test, with non-experienced horses showing more affiliative behaviors towards the familiar handler and unfamiliar persons. No significant differences were found between selected and non-selected horses in the temperament tests. However, non-selected horses were found to show significantly more affiliative behaviors towards a familiar person during a sociability test compared with selected horses. These findings suggest that the social behavior and temperament of EAS horses may not be significantly different from other available horses not selected for EAS work. Instead, these decisions may primarily reflect subjective impressions of fit. Interestingly, on measures where significant differences were identified, the horses not actively engaged in or selected for therapy were the ones that showed greater affiliative responses to familiar and unfamiliar humans. Reasons for why this may be, as well as future directions in EAS selection, are discussed.

## 1. Introduction

The inclusion of horses in healthcare, education, and adaptive sports has become increasingly common. For example, one organization, the Professional Association of Therapeutic Riding International (PATH Intl.), reports that an estimated 7900 horses are working at their affiliated centers. People are often drawn to horses because of their high levels of sociability. Practitioners who include horses in mental and behavioral healthcare treatments have long argued that therapeutic change is driven by relational components occurring during human–equine interactions [1]. More recently, equine-assisted services (EAS) have also captured the attention of the research community, with the majority of these empirical studies focused on the efficacy and effectiveness of EAS in human healthcare treatment [2]. However, a substantial knowledge gap exists regarding equine characteristics, such as temperament and behavior, that may contribute to, or help predict, a horse’s success and longevity as an assistance animal [3,4]. Data about the health and behavior of horses engaged in EAS is also needed to assess and track how participation in such activities may impact horse welfare [4].

Generally speaking, EAS horses are expected to demonstrate a calm presence throughout sessions [1,5,6,7,8,9,10,11]. Surveys suggest that from the equine-assisted practitioner’s perspective, the desired traits of EAS horses depend on the type of program being delivered, as client safety must be balanced with relational interactions between clients and horses. For example, when DeBoer [12] asked professionals working at PATH, Intl.-affiliated centers to indicate what they considered to be the most desirable traits of horses and ponies working in mental health treatment sessions, the survey respondents ranked characteristics such as “curious”, “tolerant”, “calm”, “sociable”, and “gentle” to be the overall most desirable traits. When horseback riding is the main activity component, such as in hippotherapy or adaptive riding, the importance of a horse being unflappable when they are introduced to novel stimuli or new situations is often stressed as a key criterion for selection [13].

What is less well understood is if horses selected for EAS participation are actually more likely to display these traits than horses deemed to be a poor fit for participation. As with other therapy animal programs [1,5,14], EAS tend to have high drop-out rates of animals who are deemed not to be the right fit for the program [1,5,14]. Temperament and personality differences among horses have been identified [15,16,17]. However, research investigating behavioral or temperament factors that predict EAS horse success is limited [9], and the use of validated behavioral tests and surveys is not a standardized part of horse selection in many EAS programs. Consequently, while most EAS providers believe they can select horses that will be a good fit for a program or for a specific rider, the differences between chosen and unchosen horses are not always readily apparent or reliable to others [18,19]. Therefore, in addition to survey-based studies, evaluating the behavior of EAS horses through direct observation is important to fully understand what factors predict EAS horse success. 

In the current study, we ask if behavioral differences between EAS and non-EAS horses across three sites could be detected using a battery of social behavior and temperament assessments, similar to those used in prior research [15,16]. Specifically, we focused on measures of sociability, curiosity, and calmness to evaluate key traits that have previously been reported as important selection criteria by practitioners [12]. We predict that if selection for EAS participation is effectively being made based on these preferred criteria, then we should see measurable differences in behavior and temperament between practicing EAS horses and horses not selected for work in EAS.

## 2. Materials and Methods

### 2.1. Subjects 

Thirty horses total were included in this study. For the first analysis, horses were broken into two groups: experienced (*N* = 19) horses (horses that had previously participated in at least one EAS intervention) and inexperienced (*N* = 11) horses (horses with no previous EAS experience) (see Table 1). 

For the second analysis, all 30 horses were then retrospectively sorted into two groups according to whether they were chosen/retained for future work in EAS programs (called “selected”; *N* = 17) or were not chosen/removed from future work in EAS programs (called “non-selected”; *N* = 13). Equine managers at each research site made selections according to their professional assessments of which horses were a good fit for EAS work and which horses should not start or, in some cases, continue with EAS work. This decision was made entirely at the discretion of each site’s equine manager, independent of the behavioral testing described in this study. 

Breeds, ages, and sex of the horses at the time of participation can be found in Table 1. Horses were located at one of three sites:

- Site A: PATH Intl. Premier accredited center, located in northwest United States. This site housed six selected horses and three non-selected horses; nine experienced horses and no inexperienced horses.
- Site B: A university-based equine center in the northwest United States. This site housed four selected horses and six non-selected horses; ten inexperienced horses and no experienced horses.
- Site C: University-based PATH Intl. Premier accredited center in the southwestern United States. This site housed seven selected horses, and four non-selected horses; ten experienced horses and one inexperienced horse.

### 2.2. Methods 

All horses participated in a sociability test and a series of temperament tests (all behavioral assessments). Tests took place in a standard round pen that all horses were familiar with, and horses were tested individually. In all of the tests, anytime an individual was not inside the pen with the horse or was not filming the horse, they were positioned out of sight (either behind a barrier or inside a nearby barn). Between the end of the sociability test, the start of the first temperament test, and between each temperament test, a two-minute “recess” was given in which the horse was left alone in the round pen for two minutes and could behave freely. In addition, all tests were filmed for retrospective behavioral analysis. 

#### 2.2.1. Sociability Test 

This test was eight minutes long and consisted of four phases. Each phase was a total of two minutes long, modeled after previous literature with attachment and sociability tests [20,21]. The horse was turned loose in the round pen, and a cowboy hat and a ball were placed into the center of the round pen as items that the horse could explore [22]. Familiar phase (2 min): A familiar caretaker entered the round pen and stood exactly one meter to the right of the gate. The familiar caretaker was instructed not to acknowledge, call to, or engage with the horse. If the horse approached and made contact, they were allowed to pet the horse twice before returning to neutral affect. If the horse moved away and again returned, this interaction was repeated. This was done to ensure any interactions were initiated by the horse and not the human. Unfamiliar phase (2 min): The familiar caretaker left the round pen and an unfamiliar person entered, standing exactly one meter to the left of the gate (whatever direction was opposite of where the familiar person stood). The unfamiliar person was given the same instructions as the familiar caretaker (the familiar and unfamiliar phases were counterbalanced across subjects). Alone phase (2 min): The unfamiliar person left the pen, and the horse was left alone. Preference assessment phase (2 min): Following the alone period, both the familiar and unfamiliar persons entered the pen. This time, the unfamiliar person stood one meter to the right of the gate while the familiar caretaker stood to the left. They were instructed to behave in the same way they did in their previous phases. At the end of the phase, both exited the pen. 

#### 2.2.2. Temperament tests

Three temperament tests were performed following a brief two-minute break. The first test was a brushing test, in which the horse was haltered and held by a familiar caretaker and an unfamiliar person approached the horse, stood parallel (with the unfamiliar person facing the horse’s shoulder) to the horse’s body, and began to brush the horse. The handler was told to interfere as little as possible and to give the horse a loose lead line. The test continued for one minute from the start of the brushing.

In the next temperament test, colorful, multicolored rugs were laid out on the ground around a bucket containing a preferred food item (i.e., carrots or grain). A familiar person stood with the horse at the opposite end of the pen while the experimenter put the treats into the bucket, shook the bucket to get the horse’s attention, and then set the bucket behind the rugs such that the horse had to step on the rugs to get to the bucket. The familiar person then led the horse towards the rugs, stopping and unclipping the lead rope when they were 1 m away from the bucket. Both humans stood neutrally until the horse ate the food or for two minutes. If the horse did not cross the rug to obtain the food after two minutes, the handler was instructed to gather the horse, lead them to the opposite end of the pen, and attempt the same methods again. If the horse failed to cross the rugs within two minutes for a second time, this was recorded, and the test ended.

In the final temperament test, the horse was left alone in the round pen. The experimenter approached the pen from the outside and tapped a closed umbrella against the fence to gain the horse’s attention. When the horse was looking, the experimenter opened the umbrella and tossed it into the round pen. The experimenter then left the area. The horse was filmed for one minute, beginning from the moment the umbrella was opened. 

All tests were done on the same day for each horse. Each test was later coded by two blind, independent coders according to the behavior survey (see Supplementary File S1), which was developed using a rating system from −3 to 3 and utilized validated ethograms from past studies [6,15,16,18,23,24]. 

#### 2.2.3. Statistical Analysis

Shapiro–Wilks tests indicated that there was a mix of normal and non-normal data. Data for the unfamiliar phase and the preference assessment of the sociability test and the brushing and umbrella tests had normal distributions (*p* > 0.05), and, therefore, parametric tests and Welch’s *t*-tests were used in the data analysis. Data from the familiar and alone phases of the sociability test as well as the bucket temperament test had non-normal distributions (*p* < 0.05); therefore, non-parametric analyses, Wilcoxon rank-sum tests and Wilcoxon signed-rank tests were used.

## 3. Results

### 3.1. EAS Experienced or Inexperienced Horses

**Figure 1:**
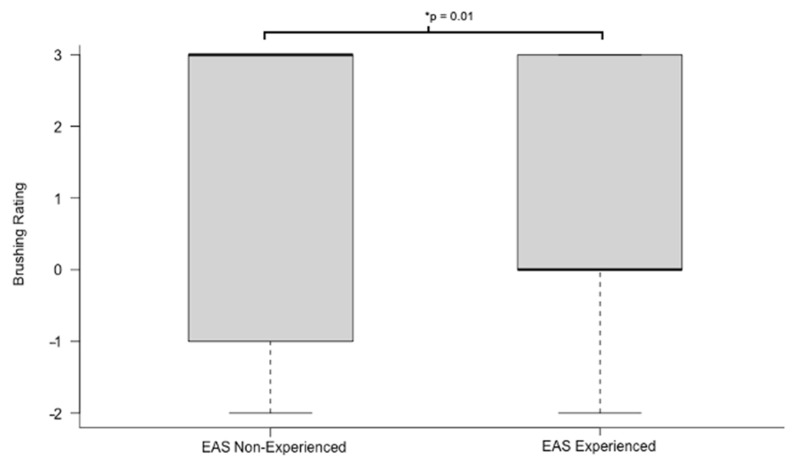
EAS experienced versus non-experienced horses’ ratings during the temperament brushing test. The bold horizontal line indicates the mean. *: Significant differences.

No differences were found between any phases of the sociability test (Wilcoxon rank-sum test, *p* > 0.05) or the umbrella or bucket temperament test (Wilcoxon rank-sum test, *p* > 0.05). However, significant differences were found between the experienced (horses that had previously participated in at least one EAS intervention) and inexperienced horses (horses that had no previous EAS experience) in the brushing temperament test (Welch’s *t*-test, *t* = 2.77, *p* = 0.01). Inexperienced therapy horses showed, on average, significantly more affiliative behaviors towards the familiar handler and unfamiliar brusher. Experienced horses, in contrast, were largely scored as behaving neutrally or slightly antagonistically towards the handler and brusher (see Figure 1). In fact, 8 of the 11 inexperienced therapy horses scored a three in this category (the highest rating, which indicates strong affiliative behaviors towards all humans) during the brushing test while only 4 of the 19 experienced horses scored a three, a finding that was statistically significant (Fisher’s exact test, *p* < 0.05). 

### 3.2. Horses Selected or Not Selected for Therapeutic Intervention

A Shapiro–Wilks test indicated that there was a mix of normal and non-normal data. Data sets for the familiar phase of the sociability test and the umbrella test had a normal distribution (*p* > 0.05); therefore, we used parametric statistics and Welch’s *t*-tests. The data for the unfamiliar, alone, and preference assessment phases of the sociability test, as well as the brush and bucket temperament tests, had non-normal distributions (*p* < 0.05) and required non-parametric analyses; therefore, Wilcoxon rank-sum tests were used. Interrater reliability was strong for all behavioral measures, with 75.8% of ratings in agreement, within 2 points of each other using the behavioral survey rating system (see supplemental material for the full scoring survey).

No differences were found in any of the temperament tests between the selected horses (horses chosen/retained for future work in EAS programs) and non-selected horses (horses not chosen/removed from future work in EAS programs) (brush: Wilcoxon rank-sum test, W = 121, *p* = 0.66; bucket: Wilcoxon rank-sum test, W = 101, *p* = 0.70; umbrella: Welch’s *t*-test, *t* = 0.040, *p* = 0.97). Nor were differences found in the alone phase, the unfamiliar phase, and the preference assessment phase of the sociability test (Wilcoxon rank-sum test, alone: W = 114, *p* = 0.90; unfamiliar: W = 102, *p* = 0.73; preference assessment: W = 112, *p* = 0.95). 

**Figure 2:**
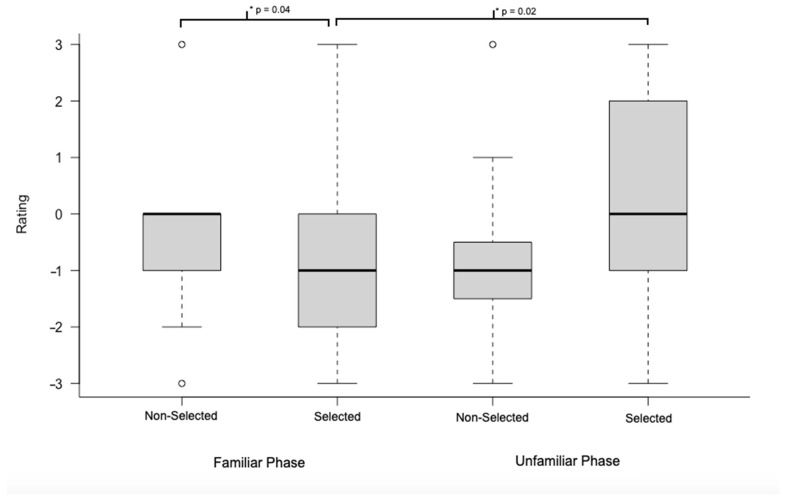
Within-group differences as well as between-group differences in the non-selected horses and selected horses are shown. The bold line represents the mean, and circles are outliers. *: Significant differences.

Significant differences were found within groups between the familiar and unfamiliar phases of the sociability test for the selected horses (Wilcoxon signed-rank test, V = 3.5, *p* = 0.02), with the selected horses being more affiliative (spent more time in proximity, approached, and/or made physical contact with the person) towards an unfamiliar person compared to a familiar handler. The same was not true of the non-selected horses, who showed no significant differences between the familiar and unfamiliar people (Wilcoxon signed-rank test, V = 17.5, *p* = 0.59). However, this lack of difference was not due to less interest in unfamiliar people on the part of the non-selected horses as both groups were equally affiliative towards an unfamiliar human (Wilcoxon rank-sum test, *p* > 0.73). Instead, this outcome was driven by the fact that non-selected horses showed greater social interest in the familiar handler. A significant difference was found between selected and unselected horses in the familiar phase of the sociability test (Welch’s *t*-test, *t* = 2.18, *p* = 0.04). The selected horses were significantly less engaged with the familiar person (looked at the person less and spent more time out of arms reach, based on coder’s ratings) compared to the non-selected horses or, conversely, the selected horses were less affiliative towards the familiar handler compared to the unfamiliar person (see Figure 2).

### 3.3. Differences between Sites

A Shapiro–Wilks test indicated that there was a mix of normal and non-normal data. Data sets for the alone and preference assessment phases of the sociability test and the rug and umbrella temperament tests had a normal distribution (*p* > 0.05); therefore, we used parametric statistics (ANOVA tests) to assess differences between sites. All other data were non-parametric, so Kruskal–Wallis tests were used. No differences were found between any of the sites (*p* > 0.05 for all); however, some trends were found in the alone (ANOVA, *p* = 0.05) and preference assessment phases (ANOVA, *p* = 0.06), with horses in Site 3 scoring generally lower in the alone phase and higher in the preference assessment phase. A slight trend (Kruskal–Wallis, *p =* 0.05) was also found in the brushing temperament, where horses at Site 2 scored higher than horses at either of the other sites.

## 4. Discussion

As with many assistance animal programs, horses considered for EAS work are not chosen or are later removed from therapeutic programs if there is a perceived lack of fit [1,5,14]. However, the selection criterion for horses involved in EAS participation varies [12], and little research has been done to evaluate if the selection process or a horse’s continued involvement in EAS is associated with consistent horse behavior and temperament traits reported desirable by practitioners in the field [9]. In the current study, we employed validated behavioral assessments to evaluate if horses selected for and/or actively participating in EAS programs significantly differed in their social behavior or temperament on some of these key traits when compared to horses not selected for EAS participation. Significant differences between EAS and non-EAS horses were not identified for the majority of the behavioral and temperament measures evaluated. However, some differences in behavior during brushing and sociability tests were identified.

Horses with no prior EAS experience engaged in more affiliative behaviors towards familiar and unfamiliar humans during brushing, whereas, on average, experienced EAS horses behaved neutrally or slightly antagonistically during brushing. In addition, horses selected for ongoing EAS participation showed a significant preference for an unfamiliar human (over a familiar handler) on sociability tests, whereas horses not selected for EAS were just as sociable towards an unfamiliar human as selected horses and showed significantly greater sociability towards the familiar human handler.

These outcomes are especially interesting because some of the most desirable traits for EAS selection, reported by professionals, include being “tolerant”, “sociable”, and “gentle” [12], and it could be argued that in the current study, the non-selected horses demonstrated some behaviors associated with greater tolerance and sociability than selected and EAS-participating horses. Such results may suggest that the EAS horse selection process could be improved by the use of standardized behavior and temperament evaluations to select horses based on their actual, instead of perceived, behavioral responses. On the other hand, it is possible that practitioners are already selecting the most successful EAS horses but may be using different or more nuanced criteria than have previously been described. For example, perhaps the most effective EAS horses are horses that have moderate levels of sociability towards a handler and high levels of sociability towards an unfamiliar human EAS participant. In this case, sociability would still be important, but EAS success might depend on a pattern of social responsiveness instead of absolute sociability scores. Such nuances may not be fully articulated or captured by a survey evaluating what traits EAS practitioners deem most important. In fact, in studies with working dogs, less social working dogs are often higher performers at their respective tasks [14,25,26]; therefore, it is possible that a more careful look at what specific aspects of sociability or degree of sociability in EAS horses predict program selection or EAS success might be a valuable area for future research.

Another consideration when looking at the data from horses with a history participating in EAS versus those with no EAS experience is that experiences during participation may lead to changes in behavior over time or when engaged with specific familiar individuals. For example, one study found that when adolescents with a fearful attachment style interacted with horses, those particular horses showed more affiliative behavior and had steadier increases in heart rates during riding compared to the horses with adolescents who had secure or preoccupied attachment styles [27]. As horses have been found to be sensitive to a human’s physiological and psychological state [28,29], these factors should be taken into consideration when evaluating the behavior of horses during behavioral assessments or during EAS sessions. Such factors may not only influence EAS outcomes but horse welfare as well, which may be reflected in behavioral shifts. Additionally, a history of potentially uncomfortable interactions during EAS participation could explain why less affiliative behavior and more agonistic behavior was observed during the brushing test in experienced versus inexperienced horses; however, more research is needed to determine if this is the case and better understand how EAS participation influences horse welfare and behavior.

In terms of the observed differences in behavior and sociability towards familiar caretakers, horses are well known to distinguish between familiar and unfamiliar people [30,31,32,33], and human–horse attachment is complex [31,34,35,36]. Horses typically have a preference for individuals that they have positive experiences with and can recognize individuals even after ten years apart [37]. EAS, such as adaptive riding, typically involve a trained volunteer horse handler and a certified EAS professional who are familiar with the horse. The handler plays an important role in monitoring the horse–client interaction, and, therefore, positive horse–human relationships have the potential to provide support and security in the presence of unfamiliar clients and during novel and sometimes unpredictable events that could occur during EAS sessions. Previous research with other species has indicated that the human-handler bond can be an important predictor of therapeutic and service animal behavior during therapeutic sessions [38,39]. However, the horse handler is typically expected to provide a neutral presence, and the horse is expected to focus on the client. Such expectations may explain why EAS horses appeared to focus more on an unfamiliar human, while non-EAS horses focused more on the familiar handler. It is also possible that stressful experiences, including during EAS sessions, could become associated with a familiar handler. In the current study, we noted that during the sociability test, some of the selected horses were highly reactive when the familiar person was in the pen with them and spent the duration of the two minutes calling to herd mates or pacing back and forth at the other end of the pen. Although past research has indicated that horses do not necessarily derive comfort from a familiar person over a stranger in stressful situations [35], it should be noted that the selected horse’s scores *improved* when an unfamiliar person was in the pen. While this could be due to temperament, prior experience, or a combination of factors, it may suggest that at least some of these horses were not able to use the presence of their familiar handler to reduce stress in this novel situation. Further research should be done to determine how a horse’s bond with a familiar handler or its attachment style may influence the selection, success, and wellbeing of EAS horses.

Finally, training methods could also influence aspects of human–horse relationships, sociability, and EAS performance. Some preliminary research in this area exists. For example, a study by Medeiros et al. [9] examined training methods with miniature horses that would be used for EAS. They found that negative reinforcement was effective in improving some, but not all, behaviors needed for EAS, while punishment was not; the findings that are in line with training studies outside of therapy practices [9,40,41,42]. Previous research has also indicated that positive reinforcement training has a positive effect on equine welfare and the human–horse bond [37,43,44,45,46,47,48]; however, the use of positive reinforcement training in therapeutic practice and its effect on equine welfare and therapy performance has not yet been examined. The results of the current study may suggest that differences between sites exist, and a lack of power may be responsible for no significant findings; however, strong trends were found in some tests. Further research could examine the impact of past experience and husbandry practices on EAS horses.

## 5. Conclusions

Despite the increasing public interest in accessing EAS, research on the selection, training, and welfare of these horses has been relatively slow to catch up in research settings [3]. Future research would benefit from more behavioral assessments of selected and active EAS horses to determine if horses with certain temperaments or preferences for familiar or unfamiliar people are more effective in the EAS environment. In addition, research examining how these behavioral differences arise, as well as possible associations with physiological markers of stress, is needed to understand the welfare implications of these findings.

## Figures and Tables

**Table 1:** Groupings based on whether the horse was experienced in EAS or not and whether the horse was selected for EAS or not. Horse age, breed, sex, and home site are also listed.

Subject	Site	Age	Breed	Sex	EAS Experience Prior to Baseline (Y/N)	Selected (S) or Non-Selected (N) for Current Analysis
1	B	18	Quarter horse	Gelding	N	N
2	A	10	Fjord/Percheron	Gelding	Y	N
3	A	14	Morgan	Gelding	Y	N
4	B	15	Quarter Horse	Gelding	N	N
5	B	7	Thoroughbred	Gelding	N	N
6	A	22	Arabian	Gelding	Y	N
7	B	16	Appaloosa	Mare	N	N
8	B	10	Arabian/Quarter Horse	Mare	N	N
9	C	10	Gypsy Vanner	Mare	N	N
10	C	10	Gypsy Vanner	Mare	Y	N
11	C	15	AQH ^1^	Gelding	Y	N
12	C	11	AQH	Gelding	Y	N
13	B	17	Quarter horse	Gelding	N	N
14	B	18	Quarter Horse	Gelding	N	S
15	B	14	Quarter Horse	Gelding	N	S
16	A	Unk ^2^	Unk	Mare	Y	S
17	A	18	Halflinger	Mare	Y	S
18	A	13	Fjord	Mare	Y	S
19	B	25	Quarter Horse	Gelding	N	S
20	A	18	Paint	Mare	Y	S
21	B	13	Quarter Horse	Mare	N	S
22	A	13	Quarter Horse	Mare	Y	S
23	A	18	Mustang	Mare	Y	S
24	C	25	AQH	Gelding	Y	S
25	C	7	AQH	Mare	Y	S
26	C	14	AQH	Gelding	Y	S
27	C	24	AQH	Gelding	Y	S
28	C	17	Fjord	Mare	Y	S
29	C	16	Arabian	Mare	Y	S
30	C	20	AQH	Gelding	Y	S

^1.^ AHQ: American Quarter Horse. ^2.^ Unknown.

## Data Availability

The data presented in this study are available on request from the corresponding author. The data are not publicly available due to the nature of the EAS work performed at the testing sites and potential exposure of sensitive information within the dataset.

## Supplementary Materials

The following are available online at https://www.mdpi.com/article/10.3390/ani11082369/s1, Supplementary File S1: EAS Behavioral Score Chart, used for coding videos in this study.
