# Supplementary material for: Horse Behavior towards Familiar and Unfamiliar Humans: Implications for Equine-Assisted Services
Source: Animals (Basel). 2021 Aug 11;11(8):2369. doi: 10.3390/ani11082369 (PMC8388774; doi:10.3390/ani11082369)
Supplement: Supplementary file 1 [file animals-11-02369-s001.zip › animals-1310088-SI.pdf]

Horse Name:_______________

Folder/Video:_______________________

<u>Sociability Test</u>

1. Familiar

Put a checkmark in the box that best describes the horse's behavior during the video (check only one box).

Horse orients toward, approaches, and makes affiliative contact (nuzzling, etc) with familiar person	A.
Horse orients toward and approaches familiar person within arm's reach, but does not initiate contact	B.
Horse orients with whole body and approaches familiar person but does not come within arm's reach	C.
No approach to familiar person, however orients head towards familiar person; foraging and/or exploration behaviors OR stands quietly	D.
Horse does not approach or orient towards familiar person; foraging and/or exploration behaviors	E.
Horse does not approach familiar person; exhibits freezing and vocalizing while standing or walking	F.
Horse does not approach familiar person; active avoidance or fearful behavior, vocalizing while trotting or cantering	G.

For "A", add 3 points
For "B," add 2 points
For "C," add 1 point
For "D," add 0 points
For "E," subtract 1 point
For "F," subtract 2 points
For "G," subtract 3 points

1. Score:_________

2. Unfamiliar

Put a checkmark in the box that best describes the horse's behavior during the video (check only one box).

Horse orients toward, approaches, and makes affiliative contact (nuzzling, etc) with unfamiliar person	A.
Horse orients towards and approaches unfamiliar person within arm's reach, but does not initiate contact	B.
Horse orients with whole body and approaches unfamiliar person but does not come within arm's reach	C.
No approach to unfamiliar person, however orients head towards unfamiliar person; foraging and/or exploration behaviors, or stands quietly	D.
Horse does not approach or orient towards unfamiliar person; foraging and/or exploration behaviors	E.
Horse does not approach unfamiliar person; exhibits freezing and vocalizing while standing or walking	F.
Horse does not approach unfamiliar person; active avoidance or fearful behavior, vocalizing while trotting or cantering	G.

For "A", add 3 points
For "B," add 2 points
For "C," add 1 point
For "D," add 0 points
For "E," subtract 1 point
For "F," subtract 2 points
For "G," subtract 3 points

2. Score:_________

3. Alone

*Put a checkmark in the box if the horse engages in the behavior **at any point** during the test. Check all that apply- you may check multiple boxes.*

Horse engages in forage and exploration, may move around in a relaxed fashion	A.
Horse moves around pen in relaxed fashion, but does not forage	B.
Horse is neutral and calm, does not move around pen	C.
Horse has increased vigilance, less relaxed, however may still engage in some foraging and exploration	D.
Stands or walks around pen, vocalizes and/or freezes	E.
Trots or canters around pen; may vocalize and/or exhibit freezing	F.

For "A," add 3 points
For "B," add 2 point
For "C," add 1 point
For "D," subtract 1 points
For "E," subtract 2 points
For "F," subtract 3 points

3. Score:________

4. Preference Assessment

Put a checkmark in the box that best describes the horse's behavior during the video (check only one box).

Horse approaches unfamiliar person first and within the first minute of the session	A.
Horse approaches unfamiliar person first but after the first minute of the session	B.
Horse approaches familiar person first and within the first minute of the session	C.
Horse approaches familiar person first but after the first minute of the session	D.
Horse orients toward either familiar or unfamiliar person or both, but does not approach either person	E.
Horse does not approach either person; freezing/ vocalizing while standing or walking	F.
Horse does not approach either person; active avoidance/fearful behavior, vocalizing while trotting or cantering	G.

For "A", add 3 points
For "B," add 2 points
For "C," add 1 point
For "D," add 0 points
For "E," subtract 1 point
For "F," subtract 2 points
For "G," subtract 3 points

4. Score:___________

5. **Brushing**

Put a checkmark in the box that best describes the horse's behavior during the video (check only one box).

Displays affiliative behavior toward person brushing and handler	A.
Displays affiliative behavior toward person brushing only	B.
Displays affiliative behavior toward handler only	C.
No reaction/netural; does not exhibit obvious pro-social behavior, vigilance, or avoidance/fear behavior	D.
Displays intermediate vigilance and/or herd seeking behavior; no other signs of avoidance/fear; may display panniculus reflex	E.
Attempts to move away from, or increase distance between, itself and person brushing; may display panniculus reflex	F.
Active avoidance/fear; attempts to move away from person and/or handler, or displays aggressive behavior in response to brushing	G.

For "A", add 3 points
For "B," add 2 points
For "C," add 1 point
For "D," add 0 points
For "E," subtract 1 point
For "F," subtract 2 points
For "G," subtract 3 points

5. Score: _________

6. **Bucket and Rug Test**

Put a checkmark in the box that best describes the horse's behavior during the video (check only one box).

Readily crosses rug without encouragement, eats food from bucket	A.
Makes contact with rug, pauses, then crosses independently	B.
Crosses rug/bucket only after encouragement	C.
Steps on and/or sniffs rug but does not cross over to obtain treat	D.
Ignores/avoids bucket and rug	E.
Spooks at rug, may cross rug but only after encouragement	F.
Actively flees from rug, does not cross	G.

For "A", add 3 points
For "B," add 2 points
For "C," add 1 point
For "D," add 0 points
For "E," subtract 1 point
For "F," subtract 2 points
For "G," subtract 3 points

6.

Score:______

7. **Umbrella**

Put a checkmark in the box if the horse engages in the behavior ***at any point*** *during the test. Check all that apply- you may check multiple boxes.*

Pushes and/or picks up umbrella and/or plays with umbrella	A.
Makes contact with umbrella	B.
Comes within a body length of umbrella	C.
Readily approaches umbrella	D.
Watches umbrella with relaxed body posture	E.
No reaction in response to umbrella	F.
Engages in exploration and/or foraging in other areas of pen while umbrella is present	G.
Blows or snorts in the direction of umbrella	H.
Startles (jumps) – For the 1st time	I.
Startles (jumps) - 2nd time (no additional checks needed for additional startles)	J.
Vocalizes (neighs, whinnies, etc)	K.
Freezes or remains in fixed position in response to umbrella	L.
Runs or flees in response to umbrella	M.

For A, add 3 points
For B add 3 points
For C, add 2 points
For D, add 1 point
For E, add 1 point
For F, add 0 points
For G, add 0 points
For H, subtract 1 point
For I, subtract 1 point
For J, subtract 1 point
For K, subtract 2 points
For L, subtract 2 points
For M, subtract 3 points

7. Score:_________

**Sociability Score:**	Add Scores 1, 2, 4, and 5	
**Boldness Score:**	Add Scores 3, 6, and 7	
